# Nemolizumab is associated with improved sleep duration and fewer nocturnal awakenings in patients with prurigo nodularis

**DOI:** 10.1016/j.jdin.2026.06.004

**Published:** 2026-06-19

**Authors:** Yoshihito Mima, Masako Yamamoto, Ken Iozumi

**Affiliations:** Department of Dermatology, Tokyo Metropolitan Police Hospital, Tokyo, Japan

**Keywords:** nemolizumab, nocturnal awakenings, prurigo nodularis, sleep disturbance numerical rating scale, sleep duration

*To the Editor:* Prurigo nodularis (PN), a debilitating condition characterized by intense pruritus, causes sleep disturbances, psychological distress, and impaired social functioning.^1^^,^^2^ For many patients, alleviating pruritus is the primary therapeutic goal.^3^, ^4^, ^5^ Nemolizumab modulates the pathogenesis of PN by inhibiting interleukin 31–mediated neuroinflammation and fibrosis.^1^, ^2^, ^3^ Clinical trials reveal significant improvements in pruritus and lesions within 16 weeks and improvements in sleep disturbance assessed using the sleep disturbance numerical rating scale (SD-NRS).^4^^,^^5^ However, real-world data on sleep-related outcomes beyond SD-NRS scores remain limited.

This retrospective study included 22 patients with refractory PN treated with nemolizumab (60 mg initially, then 30 mg monthly). After excluding 3 patients who discontinued nemolizumab by week 16 because of adverse events (asthma exacerbation, edematous erythema, and acute eczema), 19 patients (mean age: 75.2 years; 57.9% male) were finally included. Patients with concomitant atopic dermatitis were included when stable. Missing data were not imputed, with analyses based on observed cases. Peak pruritus numerical rating scale (PP-NRS) was assessed over the preceding week at weeks 0, 4, 8, 12, and 16, whereas SD-NRS, daily sleep duration, and nocturnal awakening frequency were evaluated as well at weeks 0, 4, and 16. PP-NRS and SD-NRS scores were obtained from routine outpatient assessments, whereas sleep-related measures were collected as patient-reported outcomes from retrospective recall. Our institution’s ethics committee approved the study (approval no. 25-A03).

At baseline, mean PP-NRS and SD-NRS were 8.1 and 6.2, respectively; mean sleep duration was 6 hours, with 3.1 nocturnal awakenings per night (Table I).

**Table I:** Baseline characteristics of patients with prurigo nodularis treated with nemolizumab (*n* = 19)

Category	Value
Baseline demographics	
Age (y) mean ± SD; median (IQR)	75.2 ± 13.2; 77 (63-84)
Sex	Male, 11; female, 8
PP-NRS score mean ± SD; median (IQR)	8.1 ± 1.5; 8 (7-9)
SD-NRS score mean ± SD; median (IQR)	6.2 ± 2.4; 5.5 (4.5-8.5)
Sleep duration per night (h) (mean ± SD; median [IQR])	6.0 ± 2.1; 6 (5-7.25)
Frequency of nocturnal awakenings per night (times) mean ± SD; median (IQR)	3.1 ± 2.7; 2 (1.25-5)
Comorbidities, *n* (%)	
Atopic dermatitis	3 (15.8)
Cardiovascular disease	5 (26.3)
Hypertension	7 (36.8)
Diabetes mellitus	4 (21.1)
Hyperlipidemia	4 (21.1)
Renal dysfunction	4 (21.1)
Prior treatment	
Topical corticosteroids	19 (100)
Antihistamines	19 (100)
Photo therapy	1 (5.3)
Oral corticosteroid	1 (5.3)
Oral cyclosporine	2 (10.5)
Nalfurafine hydrochloride	1 (5.3)
Difelikefalin acetate	1 (5.3)
Dupilumab	4 (21.1)

*PP-NRS*, Peak pruritus numerical rating scale; *SD-NRS*, sleep disturbance numerical rating scale.

No changes in concomitant medications occurred during nemolizumab treatment. PP-NRS improved from baseline at weeks 4, 8, 12, and 16 (from 8.1 to 3.3, 2.0, 1.9, and 1.9, respectively; all *P <* .01). SD-NRS decreased to 3.0 at week 4 and 1.8 at week 16 (both *P* < .05). Sleep duration increased to 6.9 hours at week 4 and 7.3 hours at week 16 (both *P* < .05), whereas nocturnal awakenings decreased to 1.5 at week 4 (*P* < .05) and 0.9 at week 16 (*P <* .01) (Fig. 1, *A*). Analyses were performed by using the Friedman test with Bonferroni correction. Subgroup analyses by prior dupilumab exposure yielded comparable improvements in SD-NRS, sleep duration, and nocturnal awakenings (Fig. 1, *B*) over 16 weeks, with no significant differences between the groups.

**Fig 1:**
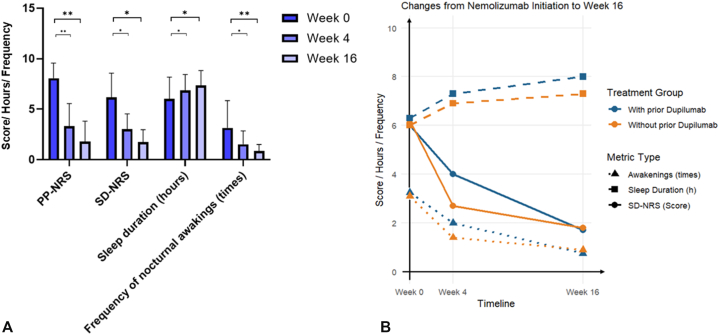
Time course of clinical and sleep-related outcomes after nemolizumab treatment in patients with prurigo nodularis, including comparisons based on prior dupilumab exposure. **A,** Time course of clinical parameters after nemolizumab treatment in patients with prurigo nodularis (PN). Significance levels are denoted as follows: ∗ *P* < .05; ∗∗ *P* < .01 (vs baseline). PP-NRS was evaluated in 19 patients at weeks 0, 4, 8, 12, and 16. SD-NRS was assessed in 12 patients, sleep duration in 18 patients, and nocturnal awakenings in 17 patients at weeks 0, 4, and 16. PP-NRS, SD-NRS, mean sleep duration, and nocturnal awakening frequency all showed significant improvement from week 4 onward. Statistical analyses were performed using the Friedman test with Bonferroni correction. The figures were generated using GraphPad Prism. **B,** Changes in sleep-related outcomes following nemolizumab treatment based on prior dupilumab exposure. SD-NRS was evaluated in 3 dupilumab-experienced and 9 dupilumab-naïve patients, sleep duration in 4 and 14 patients, respectively, and nocturnal awakenings in 4 and 13 patients, respectively. All outcomes improved similarly over time in both groups, with no significant differences at weeks 0, 4, and 16 (Mann-Whitney U test). Results are presented as line graphs showing mean values over time using R Studio. *PP-NRS*, Peak pruritus numerical rating scale; *SD-NRS*, sleep disturbance numerical rating scale.

Consistent with previous findings,^3^, ^4^, ^5^ our data demonstrate that nemolizumab significantly improves PP-NRS and SD-NRS scores in PN. Although familiar to dermatologists, these metrics may not be intuitive for other physicians or patients. This study describes nemolizumab’s effects using practical, daily-life-based sleep parameters. Improvements in sleep duration and nocturnal awakenings reflect changes in sleep behavior, complement SD-NRS-based assessments, and support the clinical utility of nemolizumab in improving PN-related sleep disturbances.

Limitations of this study include its single-center retrospective design, small sample size, and the inability to fully account for potential confounding factors affecting sleep, such as comorbidities, age, and concomitant medications. In addition, because patients were aware of receiving treatment and sleep-related outcomes were based on patient recall, the potential influence of a placebo effect cannot be excluded. Large, prospective studies are required to confirm these findings.

## Conflicts of interest

Authors Mima and Iozumi have received lecture fees from Maruho Co, Ltd, which markets nemolizumab. Author Yamamoto has no conflicts of interest to declare.
